# Entropy Gap as a Measure of Epistemic Caution in Credal Sets Generated from Data

**DOI:** 10.3390/e28060633

**Published:** 2026-06-03

**Authors:** María Isabel A. Benítez, Carlos J. Mantas, Joaquín Abellán

**Affiliations:** Department of Computer Science and Artificial Intelligence, University of Granada, 18071 Granada, Spain; miab@correo.ugr.es (M.I.A.B.); cmantas@decsai.ugr.es (C.J.M.)

**Keywords:** imprecise probabilities, reachable probability intervals, maximum entropy, epistemic uncertainty, entropy gap, credal sets, Imprecise Dirichlet Model, epsilon contamination, Non-Parametric Predictive Inference

## Abstract

Imprecise probability models generated from data represent epistemic uncertainty by replacing the precise empirical distribution with a set of compatible probability distributions. When this set is described by reachable probability intervals, the induced bounds are tight, so the represented imprecision is not inflated by unattainable interval limits. This paper studies the informational effect of this replacement through the epistemic entropy gap, defined as the difference between the maximum entropy over the induced credal set and the Shannon entropy of the empirical distribution. The gap is a differential quantity: it measures the additional uncertainty introduced by the imprecise model beyond the observed frequencies. We analyze it for three reachable interval models generated from multinomial data: the Imprecise Dirichlet Model, the ϵ-contamination model and the approximated Non-Parametric Predictive Inference model. The analysis covers its main properties, its asymptotic behavior and its role in entropy equivalent calibration of model parameters. The results show that the entropy gap offers a common informational scale for comparing how different imprecise models represent the same empirical evidence, and helps interpret the degree of caution associated with limited data reliability and with empirical distributions that may otherwise lead to overconfident uncertainty assessments.

## 1. Introduction

Imprecise probability models provide a flexible framework for representing epistemic uncertainty when the available information is scarce, noisy, partially reliable or insufficient to justify a single precise probability distribution. Instead of replacing empirical frequencies with point estimates, these models represent uncertainty through a set of compatible probability distributions, usually called a credal set [1,2]. This view is especially relevant in current uncertainty quantification, where the distinction between aleatoric and epistemic uncertainty has become central in machine learning [3]. In this paper, the term epistemic is used in a specific sense. The Shannon entropy of the empirical distribution reflects the uncertainty associated with the observed relative frequencies when they are accepted as a precise probabilistic model. By contrast, the entropy gap introduced below does not measure this empirical uncertainty again. It measures the additional increase in maximum entropy caused by replacing the empirical distribution with a credal set generated from the same data. Thus, the gap is epistemic in the sense that it is induced by the lack of commitment to a single probability distribution, or by the cautious modelling of finite, unreliable or partially trusted data. This view is also relevant for avoiding overconfident uncertainty assessments when the available data do not fully support a precise probabilistic representation. In many settings generated from data, the resulting credal set can be compactly described by lower and upper probabilities, or equivalently by a system of probability intervals.

A particularly important class of interval representations is given by reachable probability intervals [4]. Reachability guarantees that the lower and upper bounds are tight, in the sense that they can be attained by at least one probability distribution in the associated credal set. This property avoids artificial imprecision and makes reachable intervals especially useful when probability bounds are derived from empirical frequencies and later used in learning procedures.

Several relevant models generate reachable probability intervals from multinomial data. The Imprecise Dirichlet Model (IDM) introduces a cautiousness parameter whose effect decreases as the sample size increases [5,6]. The ϵ-contamination model, originally developed in robust statistics and robust Bayesian analysis, represents a fixed amount of distrust around a reference distribution and therefore preserves a non-vanishing margin of imprecision [7,8,9]. The approximated Non-Parametric Predictive Inference model for multinomial data (A-NPI-M) provides a non-parametric construction whose bounds are directly determined by the observed frequencies [10,11,12]. Although these models can be expressed through the same formal language of reachable intervals, they encode different attitudes toward the empirical information: sample-size-dependent caution, persistent distrust and parameter-free non-parametric caution.

Maximum entropy provides a natural way to quantify the total uncertainty represented by a credal set. For precise probability distributions, Shannon entropy is the classical information measure [13]. For credal sets, the maximum entropy principle extends this idea by selecting the distribution with largest entropy among all distributions compatible with the available information [14,15]. This extension preserves the interpretation of entropy as an uncertainty measure: analogously to the role of Shannon entropy in precise probability theory, maximum entropy over credal sets satisfies essential properties for measuring uncertainty under imprecision, including suitable monotonicity with respect to information content [14]. This is particularly useful for reachable probability intervals, because the maximum entropy distribution can be computed by exploiting the interval structure [14,16].

This study is motivated by the fact that different imprecise probability models may be appropriate in different data conditions. If the empirical frequencies are regarded as reliable and the main limitation is sample size, a model whose imprecision decreases with the amount of data may be preferable. If, however, the data may be affected by persistent contamination, bias, systematic noise or partial reliability, a model preserving a non-vanishing amount of caution may be more suitable. Therefore, comparing models only through their interval widths or inclusion relations may not be sufficient. A common informational criterion is needed to quantify how much additional uncertainty each model introduces with respect to the precise empirical distribution.

This observation leads naturally to a closer look at maximum entropy in credal sets generated from data. When maximum entropy is computed over such a credal set, its value combines two different sources of uncertainty. The first one is the uncertainty already present in the empirical distribution. The second one is the additional uncertainty introduced by the imprecise probability model. Distinguishing these two components is important for interpreting the degree of caution induced by each model. It is also consistent with the broader view that uncertainty measures for imprecise probabilities should have a clear behavioral interpretation and should preserve suitable information ordering properties [2,17].

We introduce an information theoretic analysis based on the epistemic entropy gap. The central idea is to compare, on a common informational scale, the uncertainty quantified by the precise empirical distribution and the maximum uncertainty represented by the credal set induced from the same data. The resulting difference measures the additional uncertainty introduced by the imprecise model beyond the observed frequencies. In this sense, the epistemic entropy gap provides a direct measure of the informational effect of replacing the precise empirical distribution with a credal set generated from the same data.

It is important to emphasize that the epistemic entropy gap is not proposed as a new total uncertainty measure for credal sets. Total uncertainty is still quantified by the maximum entropy value. The novelty lies in the differential interpretation of the gap: it measures the increase in uncertainty caused by moving from the precise empirical model to an imprecise probability model generated from the same evidence. This makes the gap complementary to existing descriptions based on interval width, structural inclusion or the maximum entropy value alone.

The analysis is developed for three models of reachable probability intervals generated from multinomial data: the IDM, the ϵ-contamination model and A-NPI-M. We study the main properties of the epistemic entropy gap, including non-negativity, monotonicity with respect to set inclusion and its upper bound in terms of the maximum possible entropy. We also analyze its behavior under the three models and its asymptotic interpretation as the sample size increases. This makes it possible to distinguish vanishing forms of caution, such as those induced by the IDM and A-NPI-M, from persistent caution, as induced by the ϵ-contamination model.

The entropy gap also supports entropy equivalent calibration between models. Rather than comparing parameters only through interval width or structural inclusion, one can compare models through the amount of additional uncertainty they induce relative to the same empirical distribution. This allows, for example, the cautiousness parameter of the IDM, the contamination parameter of the ϵ-contamination model and the parameter-free behavior of A-NPI-M to be interpreted on a common informational scale. Numerical illustrations are included to show the behavior of the measure under balanced, imbalanced and sparse frequency configurations, and to illustrate how it may be used as a diagnostic indicator of possible overconfidence in local empirical distributions, including those that arise in classification settings.

The main contributions of the paper can be summarized as follows:

- We introduce the epistemic entropy gap as a differential measure of the additional uncertainty produced when the precise empirical distribution is replaced by a credal set generated from the same data.
- We show that the gap provides information that is different from interval width, set inclusion and maximum entropy alone, because it measures the change in uncertainty relative to the empirical reference rather than total uncertainty itself.
- We establish basic properties of the gap, including non-negativity, monotonicity with respect to set inclusion, an upper bound and a characterization of the zero-gap case.
- We use the gap to compare and calibrate the IDM, the ϵ-contamination model and A-NPI-M on a common informational scale.

The remainder of the paper is organized as follows. Section 2 introduces the necessary background on credal sets, reachable probability intervals and maximum entropy. Section 3 reviews the three interval models generated from data considered in the paper. Section 4 defines the epistemic entropy gap and studies its general properties. Section 5 analyzes the behavior of this quantity for the IDM, the ϵ-contamination model and A-NPI-M. Section 6 introduces entropy equivalent calibration between models. Section 7 presents numerical illustrations. Section 8 discusses the interpretation, limitations and potential use of the proposed gap. Finally, Section 9 summarizes the main conclusions and outlines future research directions.

## 2. Background

### 2.1. Credal Sets and Probability Intervals

Let $X=\{x_{1},\ldots ,x_{K}\}$ be a finite sample space. A precise probability distribution on X is a vector

(1) $$p=(p_{1},\ldots ,p_{K}),$$

where $p_{i}=p\left(\left\{x_{i}\right\}\right)$ denotes the probability assigned to the singleton $\left\{x_{i}\right\}$, $p_{i}\geq 0$ for all i=1,…,K, and

(2) $$\sum_{i=1}^{K}p_{i}=1.$$

The set of all probability distributions on X will be denoted by $\Delta_{K}$.

A credal set P is a closed convex set of probability distributions [1,18]. This representation is useful when the available information does not justify the selection of a single precise probability distribution. Instead of committing to one element of $\Delta_{K}$, all distributions compatible with the available information are retained. In this sense, a credal set provides a natural representation of epistemic uncertainty.

Every credal set induces lower and upper probabilities for each singleton event. For each $x_{i}\in X$, these quantities are defined as

(3) $$\underline{P}\left(x_{i}\right)=\underset{p\in P}{\inf}p_{i},\;\bar{P}\left(x_{i}\right)=\underset{p\in P}{\sup}p_{i}.$$

Thus, the information associated with the singleton events can be summarized by a system of probability intervals

(4) $$I={\left\{\left[l_{i},u_{i}\right]\right\}}_{i=1}^{K},$$

where $0\leq l_{i}\leq u_{i}\leq 1$. Conversely, a system of probability intervals defines the associated credal set

(5) $$P\left(I\right)=\left\{p\in \Delta_{K}:l_{i}\leq p_{i}\leq u_{i},\;i=1,\ldots ,K\right\}.$$

Probability intervals provide a compact description of a particular class of credal sets. This representation is especially convenient for models generated from multinomial data, because the bounds can be obtained directly from observed frequencies. In this setting, different imprecise probability models can be understood as different ways of replacing the empirical distribution by a set of compatible distributions.

A general credal set can induce singleton lower and upper probabilities, but these bounds do not necessarily provide a complete description of the original credal set. In this paper, we do not start from an arbitrary credal set and then approximate it by singleton probability intervals. Instead, we restrict attention to imprecise probability models whose primary output is a system of probability intervals, and the associated credal set is precisely the set of probability distributions satisfying those interval constraints. Thus, the paper focuses on interval-generated credal sets, and more specifically on reachable probability interval models generated from multinomial data.

### 2.2. Reachable Probability Intervals

Not every system of probability intervals provides an equally meaningful representation of uncertainty. Some interval systems may include lower or upper bounds that cannot actually be attained by any probability distribution satisfying all the constraints. In that case, the intervals contain artificial imprecision because some admitted endpoint values are not compatible with the associated credal set.

The notion of reachability avoids this problem [4]. A system of probability intervals

(6) $$I={\left\{\left[l_{i},u_{i}\right]\right\}}_{i=1}^{K}$$

is said to be reachable if, for every i=1,…,K and every value $v_{i}\in \left[l_{i},u_{i}\right]$, there exists at least one probability distribution p∈P(I) such that $p_{i}=v_{i}$. In particular, the lower and upper bounds of each interval are attainable.

An equivalent way to express the reachability of interval bounds is through the following conditions. To see the connection, fix one component $p_{j}=v$. The remaining components must satisfy

(7) $$\sum_{i\neq j}p_{i}=1-v,\;l_{i}\leq p_{i}\leq u_{i},\;i\neq j.$$

Such a vector exists if and only if

(8) $$\sum_{i\neq j}l_{i}\leq 1-v\leq \sum_{i\neq j}u_{i}.$$

Requiring this feasibility condition for every $v\in [l_{j},u_{j}]$ is equivalent to checking it at the two endpoints of the interval. This gives the following conditions. For every j=1,…,K,

(9) $$\sum_{i\neq j}l_{i}+u_{j}\leq 1$$

and

(10) $$\sum_{i\neq j}u_{i}+l_{j}\geq 1.$$

These inequalities ensure that each lower and upper bound can be reached while the remaining components stay within their corresponding intervals and the total probability mass remains equal to one.

Reachability is central in this paper because it guarantees that the probability bounds are tight and do not overstate the uncertainty represented by the model. This is especially relevant when probability intervals are generated from data: if the bounds are not reachable, then the model may appear more cautious than it actually is. By restricting attention to reachable interval models, the comparison of imprecision and entropy is based on bounds that are informatively meaningful.

The three models considered later, namely the Imprecise Dirichlet Model, the ϵ-contamination model and A-NPI-M, all induce reachable probability intervals. This common feature allows them to be analyzed within the same formal framework, despite their different interpretations and motivations.

### 2.3. Maximum Entropy on Credal Sets

For a precise probability distribution $p\in \Delta_{K}$, Shannon entropy [13] is defined as

(11) $$H\left(p\right)=-\sum_{i=1}^{K}p_{i}\log p_{i},$$

with the usual convention that 0log0=0. Shannon entropy quantifies the uncertainty associated with a single probability distribution. It reaches its maximum at the uniform distribution and its minimum at degenerate distributions.

When uncertainty is represented by a credal set rather than by a single distribution, Shannon entropy cannot be applied directly to the model as a whole. A natural extension is to consider the maximum entropy value over all compatible distributions:

(12) $$H^{*}\left(P\right)=\max_{p\in P}H\left(p\right).$$

This quantity is interpreted as a measure of total uncertainty, because it accounts for the uncertainty represented by the probability distributions themselves and also for the imprecision encoded by the credal set [2,14,15].

The use of maximum entropy in imprecise probability and evidence theory is supported by strong mathematical and behavioral arguments. In particular, maximum entropy over the compatible credal set has been studied as a total uncertainty measure satisfying relevant requirements such as monotonicity with respect to information content, subadditivity and additivity under suitable independence assumptions [14,15,17]. This is important in the present work because the proposed entropy gap is meaningful only if the uncertainty measure used as reference behaves consistently with the information represented by the credal set.

For the particular case of probability intervals, the maximum entropy problem becomes

(13) $$H^{*}\left(I\right)=\max_{p\in P(I)}\left\{-\sum_{i=1}^{K}p_{i}\log p_{i}\right\}.$$

Since Shannon entropy is strictly concave and P(I) is closed and convex, the maximizing distribution is unique whenever the credal set is nonempty. For reachable probability intervals, this distribution can be obtained by a constructive allocation procedure [14,16]. The procedure starts from the vector of lower probabilities and redistributes the remaining probability mass among the smallest components, while respecting the upper bounds.

More precisely, the algorithm follows a water-filling principle. It initializes the candidate distribution at the lower bounds,

(14) $$p_{i}=l_{i},\;i=1,\ldots ,K,$$

and computes the remaining mass

(15) $$r=1-\sum_{i=1}^{K}l_{i}.$$

This mass is then allocated iteratively to the currently smallest components. More formally, let *A* be the set of indices with the smallest current value among the components that have not reached their upper bound, and let this common value be denoted by *a*. The components in *A* are increased by the same amount δ, where

(16) $$\delta =\min\left\{\min_{i\in A}\left(u_{i}-p_{i}\right),\;b-a,\;\frac{r}{\left|A\right|}\right\}.$$

Here *b* denotes the next larger current value among the components not in *A*, when such a value exists; otherwise this term is ignored. The three limiting quantities therefore correspond to the distance to an upper bound, the distance to the next current probability level and the remaining mass per active component. After the update $p_{i}\leftarrow p_{i}+\delta$ for all i∈A, the remaining mass is updated as r←r−|A|δ. The process ends when the total mass is equal to one. The resulting distribution is the unique distribution that attains the maximum entropy over P(I).

This computational property is one of the reasons why reachable probability intervals are especially useful. In evidence theory, maximum entropy over the full credal set induced by a basic probability assignment may require working with constraints over the power set of the frame of discernment, which can be computationally demanding [19,20]. By contrast, the interval-based representation restricts the computation to singleton constraints and can substantially reduce the computational burden [14,16]. Recent comparisons between belief function algorithms and interval-based algorithms show that both approaches address the same conceptual objective, although they rely on different representations of uncertainty [21].

In this paper, the maximum entropy over reachable interval credal sets plays a different but related role. It is not used only as a total uncertainty measure. It is also compared with the Shannon entropy of the empirical distribution that generated the intervals. The difference between these two quantities will be used to quantify the additional uncertainty introduced by the imprecise probability model.

In the remainder of the paper, every value of $H^{*}$ for credal sets induced by reachable probability intervals is computed using the algorithm of Abellán and Moral [14]. All numerical computations use natural logarithms and double-precision arithmetic. The allocation procedure is stopped when the normalization error satisfies $|1-\sum_{i}p_{i}|<10^{-12}$, and components closer than $10^{-12}$ to their lower or upper bounds are treated as active bounds. Entropy values reported in the tables are rounded to six decimal places, while unrounded values are used in all intermediate computations.

## 3. Data-Generated Reachable Probability Interval Models

Let $X=\{x_{1},\ldots ,x_{K}\}$ be a finite set of classes or categories. We assume that a sample of size *N* is available and denote by $n_{i}$ the number of observations assigned to class $x_{i}$. Thus,

(17) $$N=\sum_{i=1}^{K}n_{i}.$$

The empirical distribution associated with the observed frequencies is denoted by

(18) $${\hat{p}}_{i}=\frac{n_{i}}{N},\;i=1,\ldots ,K.$$

The empirical distribution provides a precise probabilistic representation of the data. However, when the available sample is limited, noisy or only partially reliable, replacing the frequency vector by a single probability distribution may lead to an overly confident representation of the available information. The three models considered in this section transform the same empirical distribution into a system of reachable probability intervals, and therefore into a credal set. In this way, each model introduces a specific form of epistemic caution around the observed frequencies.

Although the three models share the same formal representation through reachable probability intervals, they differ in the way the interval bounds are generated. The Imprecise Dirichlet Model introduces a sample-size-dependent cautiousness parameter. The ϵ-contamination model introduces a fixed amount of distrust around the empirical distribution. The A-NPI-M model provides a non-parametric correction directly determined by the observed counts. These different mechanisms will later be compared through the epistemic entropy gap.

### 3.1. The Imprecise Dirichlet Model

The Imprecise Dirichlet Model (IDM) is one of the most widely used models for inference from multinomial data under imprecise probabilities [5,6]. Instead of selecting a single Dirichlet prior, the IDM considers a set of possible priors controlled by a positive parameter *s*. In the resulting interval representation, the probability of each class $x_{i}$ is bounded by

(19) $$\left[l_{i}^{IDM},u_{i}^{IDM}\right]=\left[\frac{n_{i}}{N+s},\frac{n_{i}+s}{N+s}\right],\;i=1,\ldots ,K,$$

where s>0 is usually interpreted as a cautiousness parameter.

The width of each interval is

(20) $$u_{i}^{IDM}-l_{i}^{IDM}=\frac{s}{N+s}.$$

These intervals define a reachable system of probability intervals, as shown in [16].

Therefore, all classes receive the same amount of imprecision, independently of their observed frequencies. The parameter *s* controls the strength of the cautious component: larger values of *s* produce wider intervals and therefore larger credal sets.

For fixed *s*, the quantity s/(N+s) decreases as the sample size increases. Consequently, the imprecision induced by the IDM vanishes asymptotically:

(21) $$\lim_{N\to \infty }\left(u_{i}^{IDM}-l_{i}^{IDM}\right)=0.$$

This behavior reflects the usual interpretation that the effect of prior ignorance should become weaker as more data are observed. Thus, the IDM represents a form of epistemic caution that is strong for small samples but progressively disappears when the empirical evidence becomes large.

It is also useful to note that the empirical probability ${\hat{p}}_{i}=n_{i}/N$ always belongs to the IDM interval. Indeed,

(22) $$\frac{n_{i}}{N+s}\leq \frac{n_{i}}{N}\leq \frac{n_{i}+s}{N+s},$$

for all i=1,…,K, whenever N>0 and s>0. Hence, the precise empirical distribution is one of the distributions compatible with the IDM credal set. This property will be important when defining the epistemic entropy gap, because the gap compares the uncertainty of the empirical distribution with the maximum entropy over a credal set that contains it.

### 3.2. The ϵ-Contamination Model

The ϵ-contamination model comes from robust statistics and robust Bayesian analysis [7,8,9]. In the present data-generated setting, it can be defined around the empirical distribution as

(23) $$P_{\epsilon }=\left\{\left(1-\epsilon \right)\hat{p}+\epsilon q:q\in \Delta_{K}\right\},\;\epsilon \in \left[0,1\right].$$

The parameter ϵ controls the amount of distrust placed on the empirical distribution. When ϵ=0, the model reduces to the precise empirical distribution. When ϵ=1, the model becomes vacuous and coincides with the whole probability simplex.

For each class $x_{i}$, any distribution $p\in P_{\epsilon }$ satisfies

(24) $$p_{i}=\left(1-\epsilon \right){\hat{p}}_{i}+\epsilon q_{i},$$

for some $q\in \Delta_{K}$. Since $0\leq q_{i}\leq 1$, the induced probability intervals are

(25) $$\left[l_{i}^{\epsilon },u_{i}^{\epsilon }\right]=\left[\left(1-\epsilon \right)\frac{n_{i}}{N},\left(1-\epsilon \right)\frac{n_{i}}{N}+\epsilon \right],\;i=1,\ldots ,K.$$

Their width is

(26) $$u_{i}^{\epsilon }-l_{i}^{\epsilon }=\epsilon .$$

The following result shows that these intervals belong to the class of reachable probability intervals considered in this paper.

**Proposition** **1.** *The interval system induced by the ϵ-contamination model is reachable for every ϵ∈[0,1].*

**Proof.**  Let

(27) $$l_{i}^{\epsilon }=\left(1-\epsilon \right)\frac{n_{i}}{N},\;u_{i}^{\epsilon }=l_{i}^{\epsilon }+\epsilon ,\;i=1,\ldots ,K.$$

Using the standard reachability conditions for probability intervals, it is enough to verify that, for every j=1,…,K,

(28) $$\sum_{i\neq j}l_{i}^{\epsilon }+u_{j}^{\epsilon }\leq 1$$

and

(29) $$\sum_{i\neq j}u_{i}^{\epsilon }+l_{j}^{\epsilon }\geq 1.$$

For the first condition, we have

(30) $$\sum_{i\neq j}l_{i}^{\epsilon }+u_{j}^{\epsilon }=\left(1-\epsilon \right)\sum_{i=1}^{K}\frac{n_{i}}{N}+\epsilon =1.$$

For the second condition,

(31) $$\sum_{i\neq j}u_{i}^{\epsilon }+l_{j}^{\epsilon }=\left(1-\epsilon \right)\sum_{i=1}^{K}\frac{n_{i}}{N}+\left(K-1\right)\epsilon =1+\left(K-2\right)\epsilon .$$

Since K≥2 and ϵ≥0, we obtain

(32) $$\sum_{i\neq j}u_{i}^{\epsilon }+l_{j}^{\epsilon }\geq 1.$$

Both reachability conditions are satisfied. Therefore, the interval system induced by the ϵ-contamination model is reachable. □

Therefore, all classes receive the same interval width and this width does not depend on the sample size. This is the main difference between the ϵ-contamination model and the IDM. While the IDM becomes increasingly precise as *N* grows, the ϵ-contamination model preserves a fixed margin of epistemic caution once ϵ has been chosen.

The empirical distribution also belongs to the ϵ-contamination credal set, since it is obtained by taking $q=\hat{p}$. Equivalently, for each *i*,

(33) $$\left(1-\epsilon \right){\hat{p}}_{i}\leq {\hat{p}}_{i}\leq \left(1-\epsilon \right){\hat{p}}_{i}+\epsilon .$$

This inclusion makes the model suitable for measuring the additional uncertainty introduced with respect to the precise empirical reference.

The interpretation of this model is different from that of the IDM. The IDM reflects caution associated with finite sample information, whereas the ϵ-contamination model reflects a persistent distrust of the empirical distribution. This may be useful when the analyst wants to preserve a stable margin of caution because of possible contamination, systematic noise or partial reliability of the data source.

### 3.3. The A-NPI-M Model

Non-Parametric Predictive Inference for multinomial data provides an alternative approach that does not require the specification of a prior parameter [10,11]. In its original form, the NPI model for multinomial data does not directly lead to a closed convex credal set. For this reason, an approximated version, usually denoted by A-NPI-M, has been used in classification and related data mining procedures [12].

The A-NPI-M model generates the intervals

(34) $$\left[l_{i}^{A},u_{i}^{A}\right]=\left[\max\left\{\frac{n_{i}-1}{N},0\right\},\min\left\{\frac{n_{i}+1}{N},1\right\}\right],\;i=1,\ldots ,K.$$

These intervals were shown to be reachable by Moral-García and Abellán [22].

In contrast with the IDM and the ϵ-contamination model, A-NPI-M does not require a tuning parameter. Its bounds are completely determined by the observed counts and the sample size.

For interior frequencies, that is, when $0<n_{i}<N$, the interval takes the form

(35) $$\left[l_{i}^{A},u_{i}^{A}\right]=\left[\frac{n_{i}-1}{N},\frac{n_{i}+1}{N}\right],$$

and its width is

(36) $$u_{i}^{A}-l_{i}^{A}=\frac{2}{N}.$$

Boundary cases behave differently. If $n_{i}=0$, then

(37) $$\left[l_{i}^{A},u_{i}^{A}\right]=\left[0,\frac{1}{N}\right],$$

whereas if $n_{i}=N$, then

(38) $$\left[l_{i}^{A},u_{i}^{A}\right]=\left[\frac{N-1}{N},1\right].$$

In both boundary cases, the interval width is 1/N.

Thus, A-NPI-M also induces vanishing imprecision as the sample size increases. However, its behavior is more directly affected by the observed frequency configuration than that of the IDM or the ϵ-contamination model. In particular, classes with zero frequency may still receive positive upper probability. This feature is relevant in sparse multinomial problems and, more generally, in local frequency estimates based on small samples. Classification nodes are one possible example of such a setting, although the present paper does not develop a specific classification tree model.

As in the previous two models, the empirical distribution belongs to the A-NPI-M credal set. For each *i*, the empirical value $n_{i}/N$ lies between the lower and upper bounds:

(39) $$l_{i}^{A}\leq \frac{n_{i}}{N}\leq u_{i}^{A}.$$

Therefore, the entropy of the empirical distribution can again be used as a precise reference point for measuring the additional uncertainty induced by the imprecise model.

### 3.4. Common Structure of the Three Models

The three models considered in this paper transform the same empirical distribution into a reachable interval credal set. They all contain the empirical distribution and therefore can be compared with the precise empirical model through the entropy gap. However, they represent different attitudes toward the information provided by the data.

The IDM introduces a sample-size-dependent form of caution controlled by *s*. Its interval width is constant across classes but decreases as *N* increases. The ϵ-contamination model introduces a fixed amount of caution controlled by ϵ, independent of the sample size. A-NPI-M introduces a parameter-free correction whose width is of order 1/N, with special behavior at the boundary cases.

These differences suggest that the three models may induce different amounts of additional uncertainty even when they are generated from the same frequency vector. The epistemic entropy gap proposed in the next section provides a common informational scale for quantifying these differences.

## 4. The Epistemic Entropy Gap

### 4.1. Definition

Let $\hat{p}$ be the empirical distribution obtained from a frequency vector $\left(n_{1},\ldots ,n_{K}\right)$, and let P be a credal set generated from $\hat{p}$ by an imprecise probability model. We define the epistemic entropy gap as

(40) $$EG\left(P\right)=H^{*}\left(P\right)-H(\hat{p}),$$

where $H(\hat{p})$ is the Shannon entropy of the empirical distribution and $H^{*}\left(P\right)$ is the maximum entropy over the credal set P. Equivalently, the decomposition

(41) $$H^{*}\left(P\right)=H\left(\hat{p}\right)+EG\left(P\right)$$

separates the uncertainty already present in the empirical distribution from the additional uncertainty introduced by the imprecise model. The first term depends only on the observed frequencies, whereas the second term depends on the enlargement from the precise empirical model to the credal set. This is the reason why EG(P) is interpreted here as epistemic caution.

The quantity $H(\hat{p})$ represents the uncertainty associated with the precise empirical model, that is, the uncertainty obtained when the observed frequencies are accepted as a single probability distribution. By contrast, $H^{*}\left(P\right)$ represents the maximum uncertainty compatible with the imprecise model generated from the same data. Therefore, the entropy gap measures the increase in uncertainty produced when the precise empirical distribution is replaced by a credal set. In this sense, it quantifies the informational effect of moving from a precise data representation to an imprecise one.

The proposed quantity is different from other ways of comparing imprecise probability models. Interval width describes the local range allowed for each individual probability, and set inclusion gives a structural ordering between credal sets. The value $H^{*}\left(P\right)$ measures total uncertainty under the imprecise model. By contrast, EG(P) is a differential measure: it uses the empirical entropy as a reference and measures only the additional uncertainty induced by the credal representation.

For a fixed empirical distribution, a small value of EG(P) indicates that the imprecise model remains close, from an informational point of view, to the precise empirical representation. A larger value means that the maximum entropy admitted by the credal set is farther above the empirical entropy and closer to the upper limit logK. Its practical importance should therefore be assessed relative to the available range $\left[0,\log K-H\left(\hat{p}\right)\right]$. Thus, the entropy gap can be understood as a measure of epistemic caution.

The appropriate magnitude of this gap depends on the context in which the data are generated. If the data are reliable and representative, a small gap may be desirable. If the data may be affected by persistent contamination, systematic noise, selection bias or partial reliability, then a larger gap may be justified. In such situations, an imprecise model should not collapse too quickly to the empirical distribution, because the amount of data alone does not remove the possible unreliability of the data generating process.

The absolute magnitude of EG(P) should nevertheless be interpreted with care. The gap is directly comparable when the empirical distribution and the sample space are fixed, or when several models are generated from the same data. Comparisons across different tasks, different numbers of classes or different empirical distributions require normalization or contextual interpretation, because the maximum possible value of the gap is $\log K-H(\hat{p})$. When such cross-problem comparisons are required, one may consider the normalized quantity

(42) $$NEG\left(P\right)=\frac{EG(P)}{\log K-H(\hat{p})},$$

whenever $\log K>H(\hat{p})$. This normalized version is not the main object of the paper, but it can help interpret the relative size of the gap across different sample spaces.

From a diagnostic viewpoint, the gap is especially useful for local empirical distributions that appear highly concentrated. Such distributions may arise, for example, in classification nodes, local neighborhoods or decision-support summaries based on limited data. In these cases, a low empirical entropy may suggest a confident prediction, but the entropy gap indicates how much this confidence changes when finite-sample imprecision, unobserved classes or partial data reliability are taken into account. This interpretation is descriptive: the gap can signal possible overconfidence, but it does not by itself validate a predictive model or replace an empirical performance study.

Given a fixed frequency vector and fixed model parameters, the values

(43) $$EG(P_{IDM}),\;EG(P_{\epsilon }),\;EG\left(P_{A}\right)$$

allow us to compare how much additional uncertainty is introduced by the IDM, the ϵ-contamination model and A-NPI-M, respectively. This comparison is not only structural but also informational: it evaluates the effective change in uncertainty produced by each model relative to the precise empirical reference.

In the following, when the credal set is induced by a specific model *M* with parameter θ, we will also write

(44) $$EG_{M}\left(\hat{p},\theta \right)=H^{*}\left(P_{M}\left(\hat{p},\theta \right)\right)-H\left(\hat{p}\right).$$

For instance, θ=s for the IDM and θ=ϵ for the ϵ-contamination model. In the case of A-NPI-M, the model has no external tuning parameter and the gap depends only on the observed frequency vector and the sample size.

Finally, the epistemic entropy gap should not be interpreted as a universal criterion for selecting the “best” imprecise probability model. A larger gap indicates greater epistemic caution, but greater caution is not always preferable. The suitable amount of caution depends on the reliability of the data and on the purpose of the analysis.

### 4.2. Basic Properties

The epistemic entropy gap satisfies several basic properties that justify its interpretation as an informational measure of epistemic caution. Throughout this subsection, $\hat{p}$ denotes the empirical distribution used as reference and P denotes a credal set generated from $\hat{p}$.

**Proposition** **2** (Non-negativity)**.** *If $\hat{p}\in P$, then*

(45) EG(P)≥0.

**Proof.**  Since $\hat{p}\in P$, we have

(46) $$H^{*}\left(P\right)=\max_{p\in P}H\left(p\right)\geq H\left(\hat{p}\right).$$

Therefore,

(47) $$EG\left(P\right)=H^{*}\left(P\right)-H\left(\hat{p}\right)\geq 0.$$

□

This property is essential for the intended interpretation of the gap. When the empirical distribution belongs to the credal set, moving from the precise empirical model to the imprecise model cannot reduce the maximum uncertainty. The imprecise model may leave the entropy unchanged, but it cannot introduce a negative amount of epistemic uncertainty relative to the empirical reference.

**Proposition** **3** (Zero gap for the precise empirical model)**.** *If $P=\{\hat{p}\}$, then*

(48) EG(P)=0.

**Proof.**  If the credal set contains only the empirical distribution, then

(49) $$H^{*}\left(P\right)=H\left(\hat{p}\right),$$

and therefore

(50) $$EG\left(P\right)=H^{*}\left(P\right)-H\left(\hat{p}\right)=0.$$

□

This means that the gap vanishes when no imprecision is introduced. Hence, the precise empirical distribution provides the natural zero point for the proposed measure.

**Proposition** **4** (Monotonicity with respect to set inclusion)**.** *Let $P_{1}$ and $P_{2}$ be two credal sets generated from the same empirical distribution $\hat{p}$. If*

(51) $$P_{1}\subseteq P_{2},$$

*then*

(52) $$EG\left(P_{1}\right)\leq EG\left(P_{2}\right).$$

**Proof.**  Since $P_{1}\subseteq P_{2}$, maximizing Shannon entropy over $P_{2}$ cannot give a smaller value than maximizing it over $P_{1}$. Thus,

(53) $$H^{*}\left(P_{1}\right)\leq H^{*}\left(P_{2}\right).$$

Since both gaps are computed with respect to the same empirical entropy $H(\hat{p})$, subtracting this common value gives

(54) $$EG\left(P_{1}\right)\leq EG\left(P_{2}\right).$$

□

This property connects the entropy gap with the usual information ordering of credal sets. If one model contains another model for the same data, then the larger model cannot have a smaller entropy gap. Therefore, structural caution implies at least as much informational caution when both models are evaluated relative to the same empirical distribution.

**Proposition** **5** (Upper bound)**.** *For any nonempty credal set $P\subseteq \Delta_{K}$ containing $\hat{p}$,*

(55) $$0\leq EG\left(P\right)\leq \log K-H\left(\hat{p}\right).$$

**Proof.**  The lower bound follows from non-negativity. For the upper bound, Shannon entropy on $\Delta_{K}$ satisfies

(56) H(p)≤logK

for every $p\in \Delta_{K}$. Hence,

(57) $$H^{*}\left(P\right)\leq \log K.$$

Therefore,

(58) $$EG\left(P\right)=H^{*}\left(P\right)-H\left(\hat{p}\right)\leq \log K-H\left(\hat{p}\right).$$

□

The upper bound shows that the largest possible gap depends on how far the empirical distribution is from maximum entropy. If the empirical distribution is already uniform, then $H(\hat{p})=\log K$ and the gap must be zero for every credal set containing $\hat{p}$. If the empirical distribution is highly concentrated, then there is more room for an imprecise model to increase the maximum entropy.

**Corollary** **1.** *If $\hat{p}$ is the uniform distribution on X and $\hat{p}\in P$, then*

(59) EG(P)=0.

**Proof.**  If $\hat{p}$ is uniform, then $H(\hat{p})=\log K$. Since $H^{*}\left(P\right)\leq \log K$ and $\hat{p}\in P$, we also have $H^{*}\left(P\right)\geq H\left(\hat{p}\right)=\log K$. Hence,

(60) $$H^{*}\left(P\right)=\log K$$

and the gap is zero. □

This corollary is conceptually important. It shows that the entropy gap does not increase merely because the credal set is enlarged. If the precise empirical distribution is already maximally uncertain, an imprecise model cannot increase the maximum entropy. Thus, the gap measures the informational effect of imprecision relative to the empirical distribution, not the geometric size of the credal set alone.

**Proposition** **6** (Characterization of zero gap)**.** *Assume that $\hat{p}\in P$. Then*

(61) EG(P)=0

*if and only if $\hat{p}$ is a maximum entropy distribution in P.*

**Proof.**  If EG(P)=0, then

(62) $$H^{*}\left(P\right)=H\left(\hat{p}\right),$$

so $\hat{p}$ attains the maximum entropy value over P. Conversely, if $\hat{p}$ is a maximum entropy distribution in P, then $H^{*}\left(P\right)=H\left(\hat{p}\right)$, and therefore EG(P)=0. □

This result clarifies that a zero gap does not necessarily mean that the credal set is precise. It only means that the credal set does not contain any distribution with entropy larger than that of the empirical distribution. For example, if the empirical distribution is uniform, many non-singleton credal sets containing it will still have zero gap.

These properties confirm that the epistemic entropy gap is a differential measure, not a measure of total uncertainty. While $H^{*}\left(P\right)$ quantifies the total uncertainty of the imprecise model, EG(P) quantifies the additional uncertainty introduced relative to the empirical distribution. Its interpretation is therefore descriptive and calibrating: a larger gap means greater epistemic caution, but not necessarily a better model.

## 5. Model-Specific Analysis

The general properties of the epistemic entropy gap provide a common basis for analyzing the three models considered in this paper. Since the empirical distribution belongs to the credal set induced by each model, the corresponding gap is always non-negative. Moreover, for a fixed empirical distribution, the gap cannot exceed the difference between the maximum possible entropy, logK, and the entropy of the empirical distribution. Therefore, the amount of additional uncertainty that each model can introduce depends not only on the size of the induced credal set, but also on the position of the empirical distribution within the probability simplex.

This observation is important for the model-specific analysis. If the empirical distribution is close to uniform, then its entropy is already close to the maximum possible value, and the entropy gap must be small regardless of the imprecise model used. In contrast, if the empirical distribution is concentrated on a small number of classes, then there is more room for an imprecise model to increase the maximum entropy. Thus, the gap is expected to be more informative in imbalanced, sparse or low-sample-size configurations.

### 5.1. Epistemic Entropy Gap for the IDM

For the IDM, the epistemic entropy gap is defined as

(63) $$EG_{IDM}\left(s,N,\hat{p}\right)=H^{*}\left(P_{IDM,s}\right)-H(\hat{p}),$$

where $P_{IDM,s}$ is the credal set induced by the IDM intervals with cautiousness parameter *s*.

The parameter *s* controls the amount of imprecision introduced by the model. For fixed *N* and $\hat{p}$, larger values of *s* produce wider intervals and, therefore, larger credal sets. Since the entropy gap is monotone with respect to set inclusion, the following qualitative behavior holds:

(64) $$s_{1}\leq s_{2}\;⟹\;EG_{IDM}\left(s_{1},N,\hat{p}\right)\leq EG_{IDM}\left(s_{2},N,\hat{p}\right).$$

Thus, increasing *s* cannot reduce the additional uncertainty measured by the gap. This is consistent with the interpretation of *s* as a cautiousness parameter.

For fixed *s*, the interval width is

(65) $$u_{i}^{IDM}-l_{i}^{IDM}=\frac{s}{N+s},$$

which tends to zero as the sample size increases. Hence, along any sequence of samples for which the empirical distributions converge to a fixed limiting distribution, the IDM credal set collapses to the corresponding empirical limit. In that case, the maximum entropy over the IDM credal set converges to the Shannon entropy of the empirical distribution, and therefore

(66) $$EG_{IDM}\left(s,N,{\hat{p}}^{\left(N\right)}\right)\to 0\;\mathrm{as}\;N\to \infty .$$

This asymptotic behavior shows that the IDM induces a vanishing form of epistemic caution. The model is cautious when the sample size is small, but the additional uncertainty decreases as more observations are available. This is appropriate when the main source of epistemic uncertainty is the limited amount of data. In such a case, increasing the sample size should progressively reduce the informational difference between the precise empirical model and the imprecise model.

However, the size of the entropy gap for a fixed *N* also depends on the shape of the empirical distribution. If $\hat{p}$ is uniform, then $H(\hat{p})=\log K$ and the gap is zero, even though the IDM credal set may be non-singleton. In this case, the empirical distribution already has maximum entropy, and the imprecise model cannot increase the maximum uncertainty. By contrast, if the empirical distribution is highly concentrated, the IDM intervals may contain distributions with larger entropy than $\hat{p}$, leading to a positive gap.

Therefore, the IDM gap measures the additional uncertainty introduced by a sample-size-dependent cautiousness mechanism. It is expected to be more relevant in small samples and in empirical configurations far from uniformity, and to decrease as the amount of data grows.

### 5.2. Epistemic Entropy Gap for the ϵ-Contamination Model

For the ϵ-contamination model, the epistemic entropy gap is

(67) $$EG_{\epsilon }\left(\epsilon ,\hat{p}\right)=H^{*}\left(P_{\epsilon }\right)-H(\hat{p}),$$

where

(68) $$P_{\epsilon }=\left\{\left(1-\epsilon \right)\hat{p}+\epsilon q:q\in \Delta_{K}\right\}.$$

The parameter ϵ controls the amount of distrust placed on the empirical distribution. When ϵ=0, the model reduces to the precise empirical distribution, and therefore

(69) $$EG_{\epsilon }\left(0,\hat{p}\right)=0.$$

When ϵ increases, the credal set becomes larger. Since these credal sets are nested with respect to ϵ, we have

(70) $$\epsilon_{1}\leq \epsilon_{2}\;⟹\;P_{\epsilon_{1}}\subseteq P_{\epsilon_{2}},$$

and hence, by monotonicity of the entropy gap,

(71) $$EG_{\epsilon }\left(\epsilon_{1},\hat{p}\right)\leq EG_{\epsilon }\left(\epsilon_{2},\hat{p}\right).$$

The main difference with respect to the IDM is that the interval width is

(72) $$u_{i}^{\epsilon }-l_{i}^{\epsilon }=\epsilon ,$$

which does not depend on the sample size. Consequently, if ϵ is fixed, the induced imprecision does not vanish as *N* increases. The entropy gap may therefore remain positive even for large samples. This expresses a persistent form of epistemic caution.

This behavior is meaningful when the uncertainty is not only due to the limited amount of data. If the observations may be affected by systematic noise, contamination, selection bias or partial reliability, then increasing the sample size does not necessarily remove the reason for caution. In such situations, the ϵ-contamination model can preserve a positive informational distance between the precise empirical distribution and the imprecise representation.

As in the general case, if the empirical distribution is uniform, then

(73) $$EG_{\epsilon }\left(\epsilon ,\hat{p}\right)=0$$

for every ϵ, because the empirical distribution already reaches the maximum entropy value logK. Thus, even the vacuous case cannot increase the maximum entropy beyond the entropy of the empirical distribution. In contrast, when $\hat{p}$ is concentrated or imbalanced, increasing ϵ allows the credal set to include distributions closer to uniformity, and the gap may become larger.

At the extreme value ϵ=1, the credal set coincides with the whole probability simplex. Therefore,

(74) $$H^{*}\left(P_{\epsilon }\right)=\log K\;\mathrm{when}\;\epsilon =1,$$

and the gap becomes

(75) $$EG_{\epsilon }\left(1,\hat{p}\right)=\log K-H\left(\hat{p}\right).$$

This is the maximum possible entropy gap for a fixed empirical distribution. It corresponds to the case in which the imprecise model discards all precise commitment to the empirical frequencies and allows any probability distribution.

Thus, the ϵ-contamination gap measures the additional uncertainty introduced by a persistent distrust mechanism. Its magnitude is controlled by ϵ and reflects how much caution is retained independently of the sample size.

### 5.3. Epistemic Entropy Gap for A-NPI-M

For A-NPI-M, the epistemic entropy gap is

(76) $$EG_{A}\left(N,\hat{p}\right)=H^{*}\left(P_{A}\right)-H(\hat{p}),$$

where $P_{A}$ is the credal set induced by the A-NPI-M intervals.

Unlike the IDM and the ϵ-contamination model, A-NPI-M has no external tuning parameter. Its degree of imprecision is completely determined by the frequency vector and the sample size. For interior frequencies, the interval width is

(77) $$u_{i}^{A}-l_{i}^{A}=\frac{2}{N},$$

whereas for boundary cases the width is 1/N. Therefore, the induced imprecision is of order 1/N. Along any sequence of samples for which the empirical distributions converge to a fixed limiting distribution, the A-NPI-M credal set collapses to the corresponding empirical limit, and

(78) $$EG_{A}\left(N,{\hat{p}}^{\left(N\right)}\right)\to 0\;\mathrm{as}\;N\to \infty .$$

The epistemic caution represented by A-NPI-M is therefore also asymptotically vanishing. However, its behavior is different from that of the IDM because the intervals depend more directly on the observed counts. In particular, boundary cases receive special treatment. If a class has not been observed, that is, $n_{i}=0$, then A-NPI-M assigns it the interval

(79) $$\left[0,\frac{1}{N}\right].$$

Thus, an unobserved class is not assigned positive lower probability, but it is not completely excluded either. This feature can increase the entropy gap in sparse configurations, especially when the number of classes is large relative to the sample size.

The gap induced by A-NPI-M is therefore particularly informative in situations where the empirical distribution contains zero frequencies or very small counts. In such cases, the precise empirical model may be overly restrictive because it assigns probability zero to unobserved classes. A-NPI-M introduces a limited amount of epistemic caution by allowing those classes to receive positive probability. The entropy gap quantifies the informational effect of this correction.

As with the previous models, if the empirical distribution is uniform, then the gap is zero. More generally, the gap is bounded above by

(80) $$EG_{A}\left(N,\hat{p}\right)\leq \log K-H\left(\hat{p}\right).$$

However, because the A-NPI-M intervals shrink with *N*, the model cannot preserve a positive gap asymptotically unless the empirical distribution itself changes with *N* in a way that maintains sparse or highly imbalanced configurations.

### 5.4. Comparison of the Three Gaps

The three model-specific gaps measure the informational effect of three different forms of epistemic caution. The IDM gap is controlled by a cautiousness parameter whose effect decreases with the sample size. The ϵ-contamination gap is controlled by a fixed distrust parameter and may remain positive even for large samples. The A-NPI-M gap is parameter-free and determined directly by the frequency configuration.

For a fixed empirical distribution, the values

(81) $$EG_{IDM}(s,N,\hat{p}),\;EG_{\epsilon }(\epsilon ,\hat{p}),\;EG_{A}\left(N,\hat{p}\right)$$

provide a common informational scale for comparing the additional uncertainty introduced by the three models. This comparison is different from comparing only interval widths. Interval width is a local feature of the probability bounds, whereas the entropy gap measures the global effect of the induced credal set on the maximum uncertainty value.

The comparison is also different from structural inclusion. If one model contains another, then the gap of the larger model is at least as large, by monotonicity. However, two models may be non-comparable by inclusion and still produce ordered entropy gaps. In such cases, the entropy gap provides an additional way to compare the effective amount of epistemic caution induced by the models.

This is especially relevant for parameter calibration. For example, a value of ϵ can be chosen so that the ϵ-contamination model induces the same entropy gap as an IDM with a given value of *s*, or as A-NPI-M for a given sample size. This motivates the entropy equivalent calibration developed in the next section.

The previous analysis shows that the three models induce different forms of epistemic caution. Table 1 summarizes the qualitative behavior of the entropy gap in each case.

The table highlights that the IDM and A-NPI-M mainly represent caution associated with finite sample information, whereas the ϵ-contamination model can represent persistent caution due to contamination, bias, systematic noise or partial reliability. This distinction is useful when deciding whether uncertainty should decrease with additional data or remain present because of possible unreliability in the data generating process.

## 6. Entropy Equivalent Calibration

The epistemic entropy gap provides a common informational scale for comparing different imprecise probability models generated from the same empirical distribution. This scale can also be used for calibration. Instead of choosing model parameters by matching interval widths or by imposing structural inclusion, one may choose them by requiring that two models introduce the same amount of additional uncertainty with respect to the precise empirical model.

This idea leads to entropy equivalent calibration. Two credal sets generated from the same empirical distribution are said to be entropy equivalent when their epistemic entropy gaps coincide. In this case, both models produce the same increase in maximum entropy relative to the empirical distribution, even if their interval bounds or geometric structures are different.

**Definition** **1** (Entropy equivalent credal sets)**.** *Let $P_{1}$ and $P_{2}$ be two credal sets generated from the same empirical distribution $\hat{p}$. We say that $P_{1}$ and $P_{2}$ are entropy equivalent with respect to $\hat{p}$ if*

(82) $$EG\left(P_{1}\right)=EG\left(P_{2}\right).$$

*Equivalently,*

(83) $$H^{*}\left(P_{1}\right)=H^{*}\left(P_{2}\right),$$

*because both gaps are computed relative to the same empirical entropy $H(\hat{p})$.*

This definition does not imply that the two credal sets are equal, nor that one contains the other. It only states that, for the same empirical evidence, both models induce the same increase in maximum uncertainty. Therefore, entropy equivalence captures an informational form of equivalence rather than a geometric one.

### 6.1. Equivalent Caution Between IDM and ϵ-Contamination

Given a value of *s* in the IDM, one may define an entropy equivalent value $\epsilon^{*}$ as a solution of

(84) $$EG_{\epsilon }\left(\epsilon^{*},\hat{p}\right)=EG_{IDM}\left(s,N,\hat{p}\right).$$

This value represents the contamination level that induces the same epistemic entropy gap as the IDM for the same empirical distribution.

In this particular case, the equivalence can be made explicit. If

(85) $$\epsilon_{N}=\frac{s}{N+s},$$

then

(86) $$\left(1-\epsilon_{N}\right)\frac{n_{i}}{N}=\frac{n_{i}}{N+s}$$

and

(87) $$\left(1-\epsilon_{N}\right)\frac{n_{i}}{N}+\epsilon_{N}=\frac{n_{i}+s}{N+s}.$$

Therefore, the interval system induced by the ϵ-contamination model with $\epsilon =\epsilon_{N}$ coincides with the IDM interval system with parameter *s*. Consequently, both models induce the same credal set and hence the same maximum entropy value:

(88) $$H^{*}\left(P_{\epsilon_{N}}\right)=H^{*}\left(P_{IDM,s}\right).$$

It follows that

(89) $$EG_{\epsilon }\left(\epsilon_{N},\hat{p}\right)=EG_{IDM}\left(s,N,\hat{p}\right).$$

Thus, for the IDM and the ϵ-contamination model, the entropy equivalent contamination level is given by

(90) $$\epsilon^{*}=\frac{s}{N+s}.$$

This means that the usual width-based relationship between the two models also has an informational interpretation: it gives the value of ϵ that induces the same increase in maximum entropy as the IDM.

As a numerical illustration, consider again the sparse frequency vector

(91) $$\left(n_{1},n_{2},n_{3},n_{4}\right)=(25,5,0,0),\;N=30.$$

For the IDM with s=1, the equivalent contamination level is

(92) $$\epsilon^{*}=\frac{1}{30+1}=0.0323.$$

For this frequency vector, the IDM gives

(93) $$H\left(\hat{p}\right)=0.450561,\;H^{*}\left(P_{IDM}\right)=0.600892,$$

and therefore

(94) $$EG_{IDM}\left(1,30,\hat{p}\right)=0.150331.$$

Using ϵ=0.0323 in the ϵ-contamination model produces the same intervals, the same maximum entropy value and hence the same entropy gap:

(95) $$EG_{\epsilon }\left(0.0323,\hat{p}\right)=0.150331.$$

This example shows that, for the IDM and the ϵ-contamination model, the entropy equivalent calibration is not merely numerical. It is induced by the exact correspondence between their interval representations when ϵ=s/(N+s). This situation contrasts with the calibration involving A-NPI-M, where no single width-based equivalence is available and the entropy gap provides a genuinely global informational translation.

### 6.2. Equivalent Caution Between A-NPI-M and ϵ-Contamination

A-NPI-M has no external tuning parameter, since its degree of imprecision is determined by the observed counts and the sample size. Entropy equivalent calibration can be used to express this parameter-free caution in terms of an equivalent contamination level. We define $\epsilon^{*}$ as a solution of

(96) $$EG_{\epsilon }\left(\epsilon^{*},\hat{p}\right)=EG_{A}\left(N,\hat{p}\right).$$

Thus, $\epsilon^{*}$ represents the level of persistent contamination that would introduce the same additional uncertainty as A-NPI-M for the same empirical distribution.

This calibration is useful because A-NPI-M does not have a single global interval width. For interior frequencies, its interval width is 2/N, whereas boundary cases have width 1/N. Therefore, a width-based comparison with ϵ-contamination would depend on the class considered. By contrast, entropy equivalent calibration gives a single value $\epsilon^{*}$ that summarizes the global informational effect of the A-NPI-M credal set.

Sparse configurations make this distinction particularly relevant. If some classes are not observed, A-NPI-M still assigns them positive upper probability, which may increase the maximum entropy of the induced credal set. The corresponding value $\epsilon^{*}$ indicates how much persistent contamination would be needed to reproduce the same increase in entropy.

To illustrate this interpretation, consider the sparse frequency vector

(97) $$\left(n_{1},n_{2},n_{3},n_{4}\right)=(25,5,0,0),\;N=30.$$

The empirical distribution is

(98) $$\hat{p}=\left(0.8333,0.1667,0,0\right).$$

For this configuration, A-NPI-M gives

(99) $$H\left(\hat{p}\right)=0.450561,\;H^{*}\left(P_{A}\right)=0.673915,$$

and therefore

(100) $$EG_{A}\left(N,\hat{p}\right)=0.223354.$$

Solving

(101) $$EG_{\epsilon }\left(\epsilon^{*},\hat{p}\right)=0.223354$$

gives

(102) $$\epsilon^{*}\approx 0.0540.$$

Thus, for this frequency vector, the caution induced by A-NPI-M is informationally equivalent to an ϵ-contamination model with approximately a 5.4% persistent contamination level. This value is different from the local A-NPI-M interval widths, which are 2/N for interior frequencies and 1/N for boundary frequencies. Hence, $\epsilon^{*}$ summarizes the global informational effect of the A-NPI-M credal set rather than any single interval width.

### 6.3. Equivalent Caution Between IDM and A-NPI-M

The same idea can also be used to compare the two vanishing imprecision models. For a fixed value of *s*, IDM and A-NPI-M are entropy equivalent when

(103) $$EG_{IDM}\left(s,N,\hat{p}\right)=EG_{A}\left(N,\hat{p}\right).$$

Alternatively, since A-NPI-M has no external tuning parameter, one may search for a value $s^{*}$ such that

(104) $$EG_{IDM}\left(s^{*},N,\hat{p}\right)=EG_{A}\left(N,\hat{p}\right).$$

This value $s^{*}$ can be interpreted as the IDM cautiousness parameter that reproduces the additional uncertainty induced by A-NPI-M for the same frequency vector. This calibration is useful because the two models introduce vanishing imprecision in different ways. The IDM uses a global cautiousness parameter *s*, producing intervals of width s/(N+s) for all classes. A-NPI-M is parameter-free and its intervals depend directly on the observed counts, with different behavior for interior and boundary frequencies. Thus, entropy equivalent calibration gives a global informational comparison between the two models that is not obtained by comparing interval widths alone.

This equivalence may vary substantially with the frequency configuration. In balanced situations, both gaps may be small or even zero. In sparse or imbalanced situations, A-NPI-M may behave differently because of its special treatment of boundary counts. Consequently, the entropy equivalent value $s^{*}$ need not be constant across datasets or nodes.

To illustrate this calibration, consider again the sparse frequency vector

(105) $$\left(n_{1},n_{2},n_{3},n_{4}\right)=(25,5,0,0),\;N=30,$$

with empirical distribution

(106) $$\hat{p}=\left(0.8333,0.1667,0,0\right).$$

For this configuration, A-NPI-M gives

(107) $$EG_{A}\left(N,\hat{p}\right)=0.223354.$$

Solving

(108) $$EG_{IDM}\left(s^{*},N,\hat{p}\right)=0.223354$$

gives

(109) $$s^{*}\approx 1.54.$$

Thus, for this frequency vector, the caution induced by A-NPI-M is informationally equivalent to an IDM with cautiousness parameter approximately s=1.54. This value is specific to the observed frequency configuration and should not be interpreted as a universal translation between A-NPI-M and the IDM. It only means that, for this empirical distribution, both models induce the same increase in maximum entropy relative to the precise empirical reference.

### 6.4. Computation and Interpretation

In general, entropy equivalent parameters can be obtained by solving a one-dimensional equation. For instance, to find the contamination level equivalent to a given reference model, one solves

(110) $$f\left(\epsilon \right)=EG_{\epsilon }\left(\epsilon ,\hat{p}\right)-EG_{\mathrm{target}}\left(\hat{p}\right)=0,$$

where $EG_{\mathrm{target}}\left(\hat{p}\right)$ denotes the entropy gap induced by the model used as reference.

For the IDM and the ϵ-contamination model, monotonicity follows from the nesting of the induced credal sets with respect to their parameters. In these cases, standard root finding procedures such as bisection can be safely used for entropy equivalent calibration. For other interval models, monotonicity should be verified before using bisection; otherwise, more general one-dimensional search procedures may be required. A generic procedure is therefore as follows. First, compute the empirical entropy $H(\hat{p})$. Second, compute the target gap induced by the reference model. Third, search for the parameter value in the comparison model whose maximum entropy produces the same gap. Since the empirical entropy is common to both models, this is equivalent to matching the corresponding maximum entropy values.

This computation is feasible for reachable probability interval models because each evaluation of $H^{*}$ can be carried out using the algorithm of Abellán and Moral [14], as indicated in Section 2. Therefore, entropy equivalent calibration can be performed without solving a general nonlinear optimization problem from scratch for each parameter value.

Entropy equivalent calibration should be understood as an interpretative and methodological tool. It differs from interval width calibration, which describes only the local range allowed for each individual probability. It also differs from structural inclusion, which gives a partial ordering between credal sets but does not always apply when models are non-comparable. By contrast, entropy equivalent calibration compares models through the additional uncertainty they induce relative to the same empirical distribution.

Thus, the proposed calibration does not prescribe a universally optimal model or parameter value. Instead, it helps express different forms of epistemic caution on a common informational scale. In applications where the data are reliable, a small target gap may be appropriate; when the data may be contaminated, biased or only partially reliable, a larger target gap may better reflect the desired level of caution.

## 7. Numerical Illustrations

This section presents controlled numerical examples to illustrate the behavior of the epistemic entropy gap under representative frequency configurations. The aim is not to provide a classification benchmark or to validate predictive performance on real datasets. Rather, the examples illustrate the diagnostic role that the entropy gap may play in local empirical distributions, such as those that arise in classification nodes or other data-driven predictive settings. These examples should therefore be read as a numerical illustration of the theoretical properties rather than as an empirical evaluation of learning algorithms. All entropy values are computed using the natural logarithm.

Unless otherwise stated, we use s=1 for the IDM, a commonly used value in applications of the IDM following Walley’s recommendations [5]. For the ϵ-contamination model, following the robust contamination framework [7,8], we use ϵ=0.10 as an illustrative moderate contamination level. This value should not be interpreted as a universal choice; it represents a 10% persistent margin of distrust in the empirical distribution and is used to make the numerical comparisons transparent.

**Figure 1:**
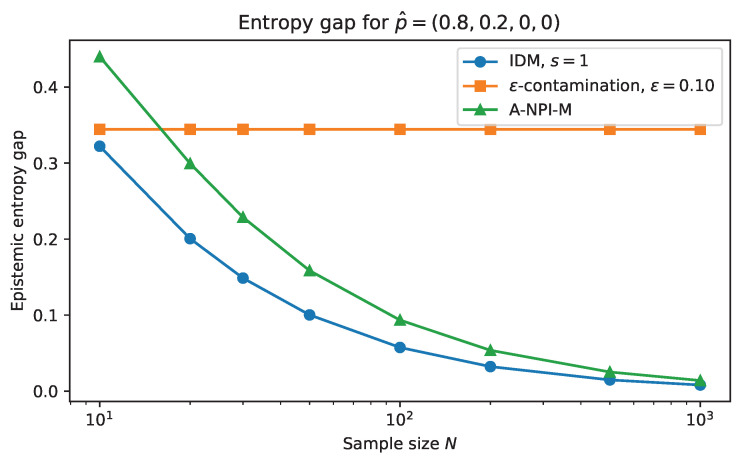
Epistemic entropy gap as a function of the sample size for the fixed empirical proportions $\hat{p}=\left(0.8,0.2,0,0\right)$. The IDM uses s=1, the ϵ-contamination model uses ϵ=0.10, and A-NPI-M has no external tuning parameter. Entropy values are computed with the natural logarithm.

Figure 1 gives a visual summary of one of the main messages of the paper. It considers the fixed empirical proportions $\hat{p}=\left(0.8,0.2,0,0\right)$ for increasing sample sizes and compares the three models under the same parameter choices used in the examples below. The figure shows that the gaps associated with the IDM and A-NPI-M decrease as the sample size grows, whereas the gap associated with a fixed ϵ-contamination level remains constant. This illustrates the difference between vanishing caution associated with finite-sample information and persistent caution associated with partial distrust of the data source.

### 7.1. Binary Case

We first consider a binary sample space

(111) $$X=\{x_{1},x_{2}\}.$$

Two frequency configurations are analyzed.

#### 7.1.1. Balanced Binary Frequencies

Let

(112) $$\left(n_{1},n_{2}\right)=(5,5),\;N=10.$$

The empirical distribution is

(113) $$\hat{p}=(0.5,0.5),$$

and therefore

(114) $$H(\hat{p})=0.693147.$$

Since the empirical distribution is uniform, it already reaches the maximum possible entropy for a binary variable. Consequently, none of the imprecise models can increase the maximum entropy value.

For the IDM with s=1, the intervals are

(115) [0.4545,0.5455],[0.4545,0.5455].

The maximum entropy distribution is

(116) $$p_{IDM}^{*}=(0.5,0.5),$$

so that

(117) $$H^{*}\left(P_{IDM}\right)=0.693147,\;EG_{IDM}=0.$$

For the ϵ-contamination model with ϵ=0.10, the intervals are

(118) [0.45,0.55],[0.45,0.55],

and again

(119) $$p_{\epsilon }^{*}=\left(0.5,0.5\right),\;H^{*}\left(P_{\epsilon }\right)=0.693147,\;EG_{\epsilon }=0.$$

For A-NPI-M, the intervals are

(120) [0.4,0.6],[0.4,0.6],

and the maximum entropy distribution is also

(121) $$p_{A}^{*}=(0.5,0.5),$$

with

(122) $$H^{*}\left(P_{A}\right)=0.693147,\;EG_{A}=0.$$

This example illustrates an important property of the epistemic entropy gap: a larger credal set does not necessarily imply a positive gap. If the empirical distribution is already maximally uncertain, the imprecise model cannot increase the maximum entropy.

#### 7.1.2. Imbalanced Binary Frequencies

Now consider

(123) $$\left(n_{1},n_{2}\right)=\left(9,1\right),\;N=10.$$

The empirical distribution is

(124) $$\hat{p}=\left(0.9,0.1\right),$$

and its entropy is

(125) $$H(\hat{p})=0.325083.$$

For the IDM with s=1, the intervals are

(126) [0.8182,0.9091],[0.0909,0.1818].

The maximum entropy distribution is

(127) $$p_{IDM}^{*}=(0.8182,0.1818),$$

with

(128) $$H^{*}\left(P_{IDM}\right)=0.474139,\;EG_{IDM}=0.149056.$$

For the ϵ-contamination model with ϵ=0.10, the intervals are

(129) [0.81,0.91],[0.09,0.19].

The maximum entropy distribution is

(130) $$p_{\epsilon }^{*}=\left(0.81,0.19\right),$$

and therefore

(131) $$H^{*}\left(P_{\epsilon }\right)=0.486223,\;EG_{\epsilon }=0.161140.$$

For A-NPI-M, the intervals are

(132) [0.8,1],[0,0.2].

The maximum entropy distribution is

(133) $$p_{A}^{*}=\left(0.8,0.2\right),$$

with

(134) $$H^{*}\left(P_{A}\right)=0.500402,\;EG_{A}=0.175319.$$

In this case, all three gaps are positive because the empirical distribution is not uniform. The imprecise models allow distributions closer to balance than the empirical distribution, increasing the maximum entropy. For the chosen parameters, A-NPI-M produces the largest gap, followed by the ϵ-contamination model and the IDM.

### 7.2. Balanced Multiclass Case

Consider now a three-class problem with frequencies

(135) $$\left(n_{1},n_{2},n_{3}\right)=(10,10,10),\;N=30.$$

The empirical distribution is

(136) $$\hat{p}=\left(\frac{1}{3},\frac{1}{3},\frac{1}{3}\right),$$

and

(137) $$H(\hat{p})=\log3=1.098612.$$

Since the empirical distribution is uniform, it already maximizes entropy on the three-dimensional simplex. Thus, for all three models,

(138) $$p_{IDM}^{*}=p_{\epsilon }^{*}=p_{A}^{*}=\hat{p},$$

and

(139) $$EG_{IDM}=EG_{\epsilon }=EG_{A}=0.$$

The corresponding interval systems are nevertheless different. For the IDM with s=1, each interval is

(140) [0.3226,0.3548].

For the ϵ-contamination model with ϵ=0.10, each interval is

(141) [0.3,0.4].

For A-NPI-M, each interval is

(142) [0.3,0.3667].

This example reinforces the distinction between interval width and entropy gap. Although the three interval systems have different widths, they all contain a uniform empirical distribution, and none of them can produce a maximum entropy value larger than log3.

### 7.3. Imbalanced Multiclass Case

We now consider a three-class imbalanced configuration:

(143) $$\left(n_{1},n_{2},n_{3}\right)=(24,5,1),\;N=30.$$

The empirical distribution is

(144) $$\hat{p}=(0.8,0.1667,0.0333),$$

and

(145) $$H(\hat{p})=0.590515.$$

For the IDM with s=1, the induced intervals are

(146) [0.7742,0.8065],[0.1613,0.1935],[0.0323,0.0645].

The maximum entropy distribution is

(147) $$p_{IDM}^{*}=(0.7742,0.1613,0.0645),$$

which gives

(148) $$H^{*}\left(P_{IDM}\right)=0.669252,\;EG_{IDM}=0.078738.$$

For the ϵ-contamination model with ϵ=0.10, the intervals are

(149) [0.72,0.82],[0.15,0.25],[0.03,0.13].

The maximum entropy distribution is

(150) $$p_{\epsilon }^{*}=(0.72,0.15,0.13),$$

and therefore

(151) $$H^{*}\left(P_{\epsilon }\right)=0.786320,\;EG_{\epsilon }=0.195805.$$

For A-NPI-M, the intervals are

(152) [0.7667,0.8333],[0.1333,0.2],[0,0.0667].

The maximum entropy distribution is

(153) $$p_{A}^{*}=(0.7667,0.1667,0.0667),$$

with

(154) $$H^{*}\left(P_{A}\right)=0.682869,\;EG_{A}=0.092354.$$

This example shows that the ϵ-contamination model may introduce a substantially larger entropy gap than the two vanishing imprecision models, even for the same frequency vector. The reason is that its fixed width allows more probability mass to be transferred toward the rare class, increasing the maximum entropy more strongly.

### 7.4. Sparse Frequencies and Unobserved Classes

Finally, consider a four-class problem with two unobserved classes:

(155) $$\left(n_{1},n_{2},n_{3},n_{4}\right)=(25,5,0,0),\;N=30.$$

The empirical distribution is

(156) $$\hat{p}=(0.8333,0.1667,0,0),$$

and

(157) $$H(\hat{p})=0.450561.$$

For the IDM with s=1, the intervals are

(158) [0.8065,0.8387],[0.1613,0.1935],[0,0.0323],[0,0.0323].

The maximum entropy distribution is

(159) $$p_{IDM}^{*}=(0.8065,0.1613,0.0161,0.0161),$$

with

(160) $$H^{*}\left(P_{IDM}\right)=0.600892,\;EG_{IDM}=0.150331.$$

For the ϵ-contamination model with ϵ=0.10, the intervals are

(161) [0.75,0.85],[0.15,0.25],[0,0.10],[0,0.10].

The maximum entropy distribution is

(162) $$p_{\epsilon }^{*}=(0.75,0.15,0.05,0.05),$$

and hence

(163) $$H^{*}\left(P_{\epsilon }\right)=0.799903,\;EG_{\epsilon }=0.349342.$$

For A-NPI-M, the intervals are

(164) [0.8,0.8667],[0.1333,0.2],[0,0.0333],[0,0.0333].

The maximum entropy distribution is

(165) $$p_{A}^{*}=(0.8,0.1333,0.0333,0.0333),$$

with

(166) $$H^{*}\left(P_{A}\right)=0.673915,\;EG_{A}=0.223354.$$

This sparse example is particularly informative. The precise empirical model assigns probability zero to the two unobserved classes. The imprecise models, by contrast, allow these classes to receive positive probability. The entropy gap quantifies the informational effect of this correction. For the chosen parameters, the ϵ-contamination model produces the largest gap because it assigns a fixed upper probability of 0.10 to each unobserved class. A-NPI-M also increases the gap by assigning upper probability 1/N to each unobserved class. The IDM introduces a smaller but still positive correction through the upper bound s/(N+s).

### 7.5. A Semi-Realistic Overconfidence Scenario

We finally consider a simple semi-realistic scenario motivated by classification nodes or local neighborhoods in which one class dominates, another class is rare and a third class has not yet been observed. The frequency vectors

(167) $$\left(n_{1},n_{2},n_{3}\right)=(9,1,0),\;(90,10,0),\;\left(900,100,0\right)$$

have the same empirical frequencies,

(168) $$\hat{p}=(0.9,0.1,0),$$

and therefore the same empirical entropy,

(169) $$H(\hat{p})=0.325083.$$

A precise predictive model based only on these frequencies would give the same highly concentrated distribution in all three cases. The entropy gap, however, reveals that the epistemic interpretation of this prediction depends on how the imprecise model uses the sample size and the unobserved class. The example is intended as a controlled diagnostic illustration. It should not be interpreted as a validated detector of overconfidence or as evidence of predictive performance without additional empirical evaluation.

Table 2 reports the gaps obtained with the same parameter choices as in the previous examples. For the IDM and A-NPI-M, the gap decreases strongly as the sample size increases, because the uncertainty associated with finite sample information vanishes. By contrast, the ϵ-contamination gap remains constant because the empirical distribution is the same and the parameter ϵ=0.10 represents a persistent distrust in the data source. This example illustrates how the entropy gap can distinguish a confident prediction that becomes epistemically stable with more data from a prediction for which a fixed contamination model still retains a substantial amount of caution. Thus, the same precise predictive entropy may hide very different degrees of epistemic stability.

This controlled example does not replace a full empirical evaluation on real datasets, but it makes explicit the diagnostic role of the gap in situations where a precise empirical distribution may appear overconfident. The same precise entropy can be associated with very different levels of epistemic caution depending on the imprecise model and on whether the source of uncertainty is interpreted as finite sample information or persistent unreliability.

### 7.6. Summary of the Numerical Values

Table 3 summarizes the numerical values obtained in the first illustrative examples. The additional semi-realistic scenario is reported separately in Table 2.

The table shows that the entropy gap is zero in balanced configurations because the empirical distribution already has maximum entropy. In imbalanced and sparse configurations, the gap becomes positive and reveals the effective increase in uncertainty induced by each imprecise model. The examples also show that the ordering of the gaps depends on the frequency configuration and on the chosen parameters. Thus, the entropy gap provides information that is not captured by interval width alone.

## 8. Discussion

The epistemic entropy gap provides a differential perspective on imprecise probability models generated from data. Maximum entropy measures the total uncertainty represented by a credal set, whereas the proposed gap measures the additional uncertainty introduced when the precise empirical distribution is replaced by an imprecise representation. Thus, the gap does not replace $H^{*}\left(P\right)$ as a total uncertainty measure; rather, it complements it by isolating the informational effect of the credal representation.

This distinction is useful because the three models studied in this paper encode different forms of epistemic caution. The IDM and A-NPI-M induce forms of imprecision that vanish as the sample size increases, although A-NPI-M is more directly affected by boundary and low-frequency cases. By contrast, the ϵ-contamination model can preserve a positive gap for fixed ϵ, even when the sample size grows. This behavior reflects persistent distrust in the data source, rather than uncertainty due only to limited sample size.

The theoretical properties and numerical examples also show that the gap should not be interpreted as a measure of the geometric size of the credal set. It depends on whether the imprecise model allows distributions with higher entropy than the empirical one. In particular, when the empirical distribution is uniform, the gap is zero for every credal set containing it, because the empirical entropy already reaches its maximum value. This is a structural limitation of the indicator: a zero gap does not imply absence of imprecision. It only means that the credal set does not increase the maximum entropy relative to the empirical reference. In such cases, complementary descriptors, such as interval widths or set inclusion relations, may still be needed when the geometry of the credal set is relevant.

The interpretation of the gap also depends on the assumed reliability of the data. If the data are reliable and representative, a small gap may be desirable, because introducing too much additional uncertainty may lead to an unnecessarily conservative model. If the data are noisy, contaminated, biased or only partially reliable, a larger gap may be appropriate. Therefore, a larger gap should not be viewed as universally better; it indicates greater epistemic caution, whose suitability depends on the application.

This idea is relevant for predictive models in artificial intelligence. In classification, a low-entropy predictive distribution is often interpreted as a confident decision. However, this confidence may be misleading when the available evidence is scarce, noisy or unreliable. The entropy gap can act as an additional diagnostic quantity: a low predictive entropy together with a small gap suggests a confident and epistemically stable prediction, whereas a low predictive entropy combined with a large gap may reveal possible overconfidence.

Table 4 summarizes this qualitative interpretation. The table is not intended as a fixed decision rule, since the thresholds defining low and high values depend on the number of classes, the application domain and the desired level of caution.

This reading suggests potential applications in uncertainty aware classification, including abstention mechanisms, human review triggers, robust calibration procedures and the detection of overconfident predictions. The semi-realistic example in Section 7 illustrates this diagnostic role: the same precise predictive entropy may correspond to different levels of epistemic stability depending on the imprecise model and on the interpretation of the data source.

The proposed entropy equivalent calibration follows naturally from this interpretation. Rather than choosing model parameters only by matching interval widths or by imposing structural inclusion, one can compare models through the amount of additional uncertainty they induce relative to the same empirical distribution. This provides an interpretable way to translate one form of epistemic caution into another.

Overall, the entropy gap should be understood as a descriptive and calibrating tool rather than as a prescriptive model selection rule. It does not identify a universally optimal model, but it helps quantify how much additional uncertainty a model introduces and how this amount relates to assumptions about sample size, data reliability and possible overconfidence. In this sense, it provides a bridge between precise empirical uncertainty quantification and imprecise probability models generated from data.

## 9. Conclusions

This paper introduced the epistemic entropy gap as a differential measure for credal sets generated from data. The proposed quantity is defined as the difference between the maximum entropy over the induced credal set and the Shannon entropy of the empirical distribution. It is not intended to replace maximum entropy as a measure of total uncertainty. Instead, it measures the additional uncertainty introduced when the precise empirical distribution is replaced by an imprecise representation through reachable probability intervals.

The analysis focused on three relevant models: the Imprecise Dirichlet Model, the ϵ-contamination model and A-NPI-M. The results show that the epistemic entropy gap provides a common informational scale for comparing different forms of epistemic caution. For fixed parameters, the gap vanishes asymptotically for the IDM and A-NPI-M, reflecting their sample-size-dependent behavior. By contrast, it may remain positive for the ϵ-contamination model, which represents a persistent form of distrust around the empirical distribution.

The theoretical properties and numerical illustrations show that the gap is not merely a measure of interval width or credal set size. It depends on the empirical frequency configuration and becomes especially informative in imbalanced or sparse settings, where imprecise models may allow distributions with substantially higher entropy than the empirical one. Thus, the magnitude of the gap should be interpreted in relation to the reliability of the data: small gaps may be appropriate for reliable data, whereas larger gaps may be justified under contamination, bias or partial reliability.

The proposed gap also supports entropy equivalent calibration between models, allowing different forms of caution to be compared through the amount of additional uncertainty they induce. At the same time, the analysis identifies an important limitation: a zero entropy gap does not necessarily imply that the induced credal set is precise, since it may also occur when the empirical distribution already has maximum entropy. In such cases, the gap should be complemented with other descriptors, such as interval widths or inclusion relations, when the geometric size of the credal set is also relevant.

Future work may study the use of the entropy gap in classification procedures, its behavior in high-dimensional multinomial problems and graphical tools for analyzing how the gap changes with sample size, class imbalance and model parameters. The semi-realistic overconfidence scenario considered in the numerical section suggests that the gap may be useful as a diagnostic quantity, but this role should be evaluated in real classification tasks, especially under noise, contamination or rare-class configurations. Extensions to more general credal sets would also require efficient procedures for computing maximum entropy.

## Figures and Tables

**Table 1:** Qualitative behavior of the epistemic entropy gap for the three interval models generated from data.

Model	Behavior of the Epistemic Entropy Gap	Type of Caution
IDM	For fixed *s*, the gap tends to vanish as the sample size increases.	Sample size dependent caution.
A-NPI-M	The gap also tends to vanish with *N*, but is sensitive to zero or very small frequencies.	Data dependent, parameter-free caution.
ϵ-contamination	For fixed ϵ>0, the gap may persist even for large samples.	Persistent distrust of the data source.

**Table 2:** Epistemic entropy gap in a semi-realistic overconfidence scenario for the IDM with s=1, the ϵ-contamination model with ϵ=0.10, and A-NPI-M. Entropy values are computed with the natural logarithm and are expressed in nats.

Frequencies	$H(\hat{p})$	${\mathrm{EG}}_{\mathrm{IDM}}$	${\mathrm{EG}}_{\epsilon }$	${\mathrm{EG}}_{A}$
(9,1,0)	0.325083	0.275083	0.292838	0.313949
(90,10,0)	0.325083	0.052327	0.292838	0.054942
(900,100,0)	0.325083	0.007576	0.292838	0.007802

**Table 3:** Empirical entropy, maximum entropy and epistemic entropy gap for the numerical examples under the same parameter choices used in Section 7: s=1 for the IDM and ϵ=0.10 for the ϵ-contamination model. Entropy values are computed with the natural logarithm and are expressed in nats.

Frequencies	Model	$H(\hat{p})$	$H^{*}$	EG
(5,5)	IDM	0.693147	0.693147	0.000000
	ϵ-contamination	0.693147	0.693147	0.000000
	A-NPI-M	0.693147	0.693147	0.000000
(9,1)	IDM	0.325083	0.474139	0.149056
	ϵ-contamination	0.325083	0.486223	0.161140
	A-NPI-M	0.325083	0.500402	0.175319
(10,10,10)	IDM	1.098612	1.098612	0.000000
	ϵ-contamination	1.098612	1.098612	0.000000
	A-NPI-M	1.098612	1.098612	0.000000
(24,5,1)	IDM	0.590515	0.669252	0.078738
	ϵ-contamination	0.590515	0.786320	0.195805
	A-NPI-M	0.590515	0.682869	0.092354
(25,5,0,0)	IDM	0.450561	0.600892	0.150331
	ϵ-contamination	0.450561	0.799903	0.349342
	A-NPI-M	0.450561	0.673915	0.223354

**Table 4:** Qualitative interpretation of predictive entropy and epistemic entropy gap in classification.

$H(\hat{p})$	EG(P)	Interpretation
Low	Low	Confident and epistemically stable prediction
Low	High	Possibly overconfident prediction
High	Low	Intrinsic ambiguity in the precise prediction
High	High	High total uncertainty and strong epistemic caution

## Data Availability

No empirical datasets were used in this study. The numerical results are based on synthetic frequency configurations generated for illustrative purposes, and all configurations needed to reproduce the calculations are reported in the manuscript.
